# Sequential super-stereotypy of an instinctive fixed action pattern in hyper-dopaminergic mutant mice: a model of obsessive compulsive disorder and Tourette's

**DOI:** 10.1186/1741-7007-3-4

**Published:** 2005-02-14

**Authors:** Kent C Berridge, J Wayne Aldridge, Kimberly R Houchard, Xiaoxi Zhuang

**Affiliations:** 1 Department of Psychology, University of Michigan, Ann Arbor, USA; 2 Department of Neurology, University of Michigan, Ann Arbor, USA; 3 Wayne State University Medical School, Detroit, USA; 4 Department of Neurobiology, Pharmacology, and Physiology, University of Chicago, Chicago, USA

## Abstract

**Background:**

Excessive sequential stereotypy of behavioral patterns (sequential super-stereotypy) in Tourette's syndrome and obsessive compulsive disorder (OCD) is thought to involve dysfunction in nigrostriatal dopamine systems. In sequential super-stereotypy, patients become trapped in overly rigid sequential patterns of action, language, or thought. Some instinctive behavioral patterns of animals, such as the syntactic grooming chain pattern of rodents, have sufficiently complex and stereotyped serial structure to detect potential production of overly-rigid sequential patterns. A syntactic grooming chain is a fixed action pattern that serially links up to 25 grooming movements into 4 predictable phases that follow 1 syntactic rule. New mutant mouse models allow gene-based manipulation of brain function relevant to sequential patterns, but no current animal model of spontaneous OCD-like behaviors has so far been reported to exhibit sequential super-stereotypy in the sense of a whole complex serial pattern that becomes stronger and excessively rigid. Here we used a hyper-dopaminergic mutant mouse to examine whether an OCD-like behavioral sequence in animals shows sequential super-stereotypy. Knockdown mutation of the dopamine transporter gene (DAT) causes extracellular dopamine levels in the neostriatum of these adult mutant mice to rise to 170% of wild-type control levels.

**Results:**

We found that the serial pattern of this instinctive behavioral sequence becomes strengthened as an entire entity in hyper-dopaminergic mutants, and more resistant to interruption. Hyper-dopaminergic mutant mice have stronger and more rigid syntactic grooming chain patterns than wild-type control mice. Mutants showed sequential super-stereotypy in the sense of having more stereotyped and predictable syntactic grooming sequences, and were also more likely to resist disruption of the pattern en route, by returning after a disruption to complete the pattern from the appropriate point in the sequence. By contrast, wild-type mice exhibited weaker forms of the fixed action pattern, and often failed to complete the full sequence.

**Conclusions:**

Sequential super-stereotypy occurs in the complex fixed action patterns of hyper-dopaminergic mutant mice. Elucidation of the basis for sequential super-stereotypy of instinctive behavior in DAT knockdown mutant mice may offer insights into neural mechanisms of overly-rigid sequences of action or thought in human patients with disorders such as Tourette's or OCD.

## Background

Overly rigid sequential patterns of movement and thought characterize several human brain disorders involving dysfunction in basal ganglia systems (i.e. dopamine nigrostriatal projections to the neostriatum and related brain structures). For example, pathological repetitions of spoken words in Tourette's syndrome, and the tormenting habits and thoughts of obsessive-compulsive disorder (OCD), involve overly rigid sequential patterns of action, language or thought [1-9], which in part may be influenced by genetic factors [10-13].

Normal sequential patterns of action, language and thought also have been suggested to depend on proper basal ganglia function [14,15]. For example, Marsden proposed that "The sequencing of motor action and the sequencing of thought could be a uniform function carried out by the basal ganglia" [15], and a variety of computational models have been proposed to carry out the general sequencing functions of basal ganglia [16-19]. According to this view, basal ganglia systems evolved originally to coordinate syntactic patterns of instinctive movements, and were extended subsequently by natural selection to participate in sequencing cognitive and linguistic functions as well.

Almost all behavior is sequential, so what do we mean by 'syntactic sequence'? In the simplest terms, a syntactic sequence is one that follows normative rules that determine the temporal progression of its elements and impart a lawful predictability to the sequence as a whole [14,20,21]. Human language has real syntax, as the prototypical example, complete with recursive generative rules [14,21,22]. But other behavior can be described as having properties of syntax too, if the behavioral flow is governed by lawful sequential patterns that follow normative rules to produce a complex serial order [14,20,23-26].

**Figure 1:**
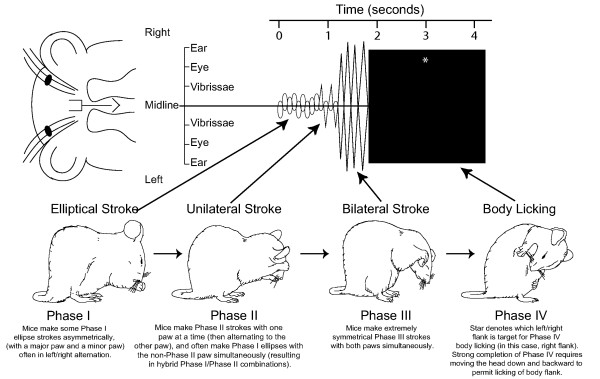
***Prototypical syntactic grooming chain pattern. ***Choreograph shows mouse movements of the left/right paws over the face (time proceeds from left to right). Lines deviating above/below the horizontal axis show the trajectory height of left/right paws. Large black box denotes bout of body licking, and placement of asterisk in box shows which left/right side flank was chosen by the mouse to initiate body licking. *Phase I*: series of ellipse-shaped strokes tightly around the nose. Left and right paws often take alternating turns as the major/minor trajectory. *Phase II*: series of unilateral strokes, each made by one paw, that reach up the mystacial vibrissae to below the eye. Mice often make hybrid Phase I/II strokes, in that one paw makes a Phase II unilateral stroke while the remaining paw makes a smaller Phase I type ellipse. *Phase III*: series of bilateral strokes made by both paws simultaneously. Paws reach back and upwards, ascending usually high enough to pass over the ears, before descending together over the front of the face. *Phase IV (strong or classic form)*: sustained bout of body licking, preceded by postural cephalocaudal transition to move mouth and tongue from facial and paw grooming to body grooming. Mouse-typical pattern modified from Berridge (1990). See Additional movie file 1 for examples of syntactic grooming chains by *DAT-KD *mutant mice.

Neuroethological studies of natural behavior in animals have shown that neostriatum, substantia nigra, and their connecting dopamine projections are critical to sequential stereotypy for complex serial patterns of instinctive behavior [26-35]. In particular, a complex fixed action pattern displayed spontaneously by rodents during grooming behavior, called a syntactic grooming chain, has been exploited by neuroethological studies that point to basal ganglia systems as the controlling neural mechanisms for the stereotypy of complex sequential patterns [27,28,36]. A syntactic chain is a 4-phase series of up to 25 elements, each phase containing recursive iterations of its characteristic element (Figure 1; see Additional movie file 1). This syntactic sequence occurs spontaneously during grooming behavior of most rodents. Mice, rats, gerbils, hamsters, guinea pigs, ground squirrels and other species all have their own signature patterns of syntactic chains, with different details, but all follow the syntactic 4-phase rule [37]. In one squirrel species (*Spermophilus beecheyi*), syntactic chains have been even further ritualized into a stereotyped display, and adapted for territorial communicative use [38]. As is typical of fixed action patterns, no two syntactic chains may be absolutely identical, but they are highly similar, stereotyped, and easily recognized, and always follow the same serial patterning rule [39,40]. Thus syntactic grooming chains are complex multi-component patterns that are sequentially stereotyped, and capable of interacting with evolutionary selection pressures that alter the genotype to modulate behavioral patterns. They represent precisely the sort of sequencing function that ancestral basal ganglia systems might originally have evolved to perform [2,9,14,15,24,29-31].

The firing of some basal ganglia neurons in neostriatum and in substantia nigra codes the serial pattern of syntactic grooming chains as an entire sequence in rats [27,28]. In addition, the integrity of basal ganglia neurons is necessary for normal sequential stereotypy of the instinctive pattern. For example, after lesions of neostriatum, rats lose the ability to complete the 4-phase pattern properly (especially after lesions of anterior dorsolateral neostriatum, which contains the neurons that particularly code the syntactic pattern), even though the lesions do not impair constituent grooming movements [27,36]. Similar deficits in grooming syntax are caused by disruption of dopamine neurotransmission in mice lacking dopamine D1 receptors [41], and in normal rats with neostriatal dopamine depletion caused by 6-hydroxydopamine lesions of nigrostriatal projections [42].

Brain lesions that *disrupt *behavioral sequences indicate a potential sequencing function for the targeted structures. However, factors besides sequencing loss may contribute to disrupted serial patterns after lesions. An alternative and stronger proof for dopamine mediation of action syntax would be to demonstrate *enhanced *stereotypy of behavioral sequences, by boosting nigrostriatal dopamine neurotransmission. Enhanced sequential stereotypy would be reflected if the complex serial pattern as a whole entity became more sequentially rigid or persistent. Indeed, in rats, pharmacological boosting by dopamine D1 agonists administered systemically or into brain ventricles produces sequential super-stereotypy of syntactic grooming chains [43-45]. In a state of sequential super-stereotypy, the stereotyped pattern becomes even more predictable than normal, which is evident as an increase in the probability that all four phases will be completed in syntactic order [43,44]. Such rigidity of complex multiple-phase sequences contrasts with simpler repetition stereotypies (e.g., associated with D2 receptor activation), in which the same movement is repeated over and over again [46-50].

In human pathologies such as Tourette's or OCD, complex sequential super-stereotypy often occurs spontaneously in human patients. If sequential super-stereotypy of complex instinctive behavior sequences is to serve as a model of human disorders involving sequential super-stereotypy, it ought to be able to occur spontaneously in some individual animals too. In addition, it should possess features of compulsive behavioral sequences.

Compulsive behavior may have several features, including both *perseverative tendencies *and *more rigid sequences of entire serial patterns*. To date, prior genetically-modified mouse models of spontaneous compulsive behavior have successfully captured the perseverative feature, but it is not yet clear whether these animal models also share the exaggerated serial pattern feature of compulsive behavior. For example, the *Hoxb8*^*lox *^mutant model has been reported to exhibit OCD-like increased persistence of self-directed grooming and body-licking, and even mutual grooming of other mice [13,51,52]. Similarly, the *D1CT *mutant mouse, caused by transgenic potentiation of D1-associated brain circuits, shows OCD-like persistence of grooming, as well as persistence of other behaviors such as digging, climbing, and tics [3,53-57]. However, it is unknown whether these or any other animal models also show excessively rigid sequences, in the sense of a stronger multi-element and rule-governed sequence that becomes more rigid as a single complex pattern. For modeling the serial rigidity feature of OCD or Tourette's, an animal model is needed that spontaneously produces an overly-rigid and serially-complex sequence of behavior, such as a syntactic grooming chain.

Here we show that this serial pattern feature of sequential super-stereotypy indeed appears spontaneously without drugs in *DAT-KD *mutant mice with genetic knockdown of the dopamine transporter (DAT) [58]. *DAT-KD *mutant mice have 10% normal DAT expression in dopamine neurons [58], which impairs synaptic re-uptake of dopamine, resulting in elevated (170%) levels of extracellular dopamine in neostriatum (wild-type mice = 100%) [58]. *DAT-KD *mutant mice show other behavioral evidence for high levels of dopamine activation. They tend to be hyperactive, to walk in perseverative straight paths, and to over-pursue certain incentive stimuli [58-60]. The question asked in the present study was whether these mutant mice would also show sequential super-stereotypy in their syntactic chains – that is, do they have excessively rigid serial patterns of instinctive grooming behavior?

## Results

### Syntactic chains

**Figure 2:**
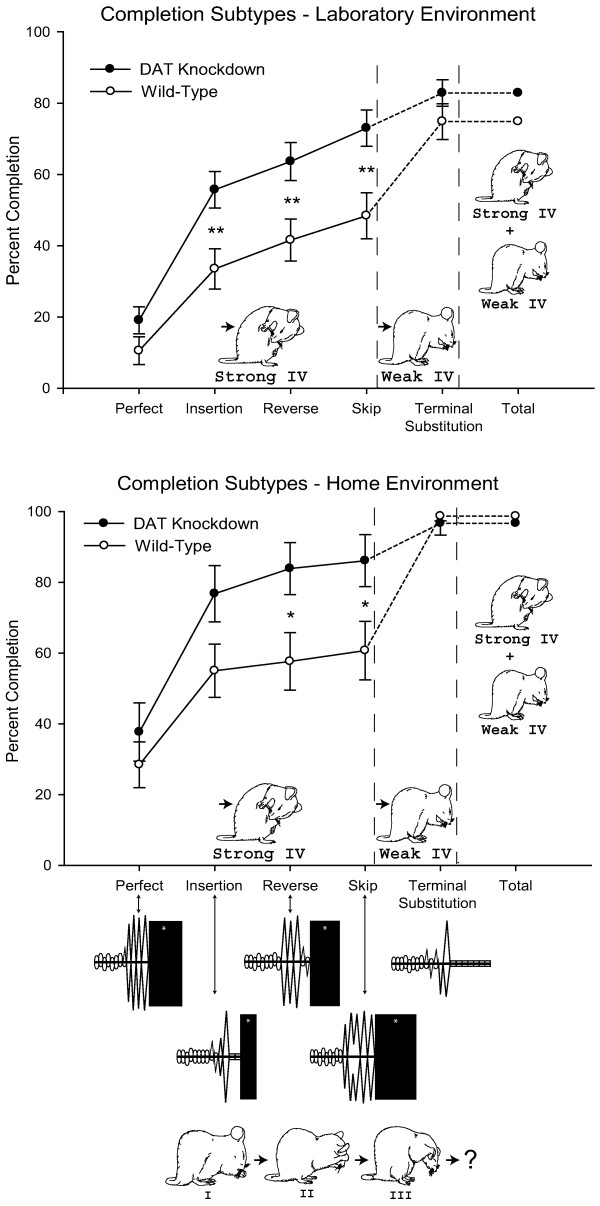
***Sequential super-stereotypy of syntactic pattern. ***Cumulative rates of full pattern completion by *DAT-KD *mutant (dark symbols) and wild-type mice (open symbols) for each type of syntactic chain (Perfect, Insertion of unpredicted component, Phase Reversal, Phase Skip, Substitution of paw lick for Terminal Phase IV component). Choreographs at bottom show example for each type of syntactic chain. Mutant mice have higher rates of syntactic completion for all forms of the chain that terminate in the strong form of Phase IV, body licking, which characterizes the prototypical Phase IV for all rodents. Wild-type mice use a weak form of Phase IV (paw lick substitution) to terminate a substantial proportion of their syntactic chains. All mice show less pattern completion when grooming in the laboratory (top) than when grooming in their home cage (bottom), but mutant mice show more rigid sequential patterns than wild-type mice while grooming in both environments. * p < 0.05; ** p < 0.01.

**Figure 3:**
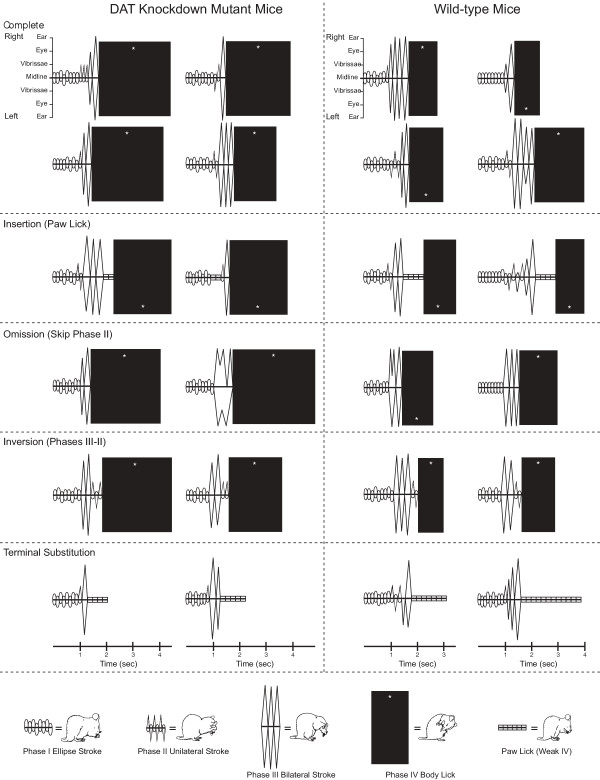
***Sample choreographs of actual syntactic chains. ***Both mutant mice and wild-type mice emit every type of syntactic chain described in the text (Perfect, Insertion of unpredicted component, Phase Reversal, Phase Skip, and Substitution of paw lick for Terminal Phase IV).

Hyper-dopaminergic mutant mice and wild-type control mice each generated syntactic chains of grooming as described above (Figures 1, 2 &3). Syntactic grooming chains by *DAT-KD *mice had virtually all the typical features of wild-type chains and of syntactic chains previously reported for outbred mice and D1 receptor knockout mice [37,41] (Figures 1 &3; see Additional movie file 1).

### Syntactic grooming chains

The sequential pattern of a syntactic grooming chain contains up to 25 movements serially combined into 4 syntactic or rule-governed phases that form one chain pattern [61] (Figure 1; see Additional movie file 1). Each of the 4 phases contains recursive repetitions of its particular component movement.

Phase I consists of 5–10 rapid elliptical forepaw strokes made with both paws simultaneously over the nose and mystacial vibrissae. In mice, Phase I ellipses are often slightly asymmetrical and alternating, in the sense that the 'major paw' makes a slightly larger stroke than the 'minor paw' [37]. Typically, the major/minor role alternates over successive Phase I strokes between left and right paws. The entire Phase I lasts for about one second.

Phase II is short (0.25 s) and consists of 1–4 unilateral or highly asymmetrical strokes made by one forepaw. The unilateral stroke is typically of small or medium amplitude ascending to about the level of the eye. In mice, the other paw not participating in the Phase II stroke often makes a smaller Phase-I ellipse-type stroke simultaneously [37,41]. Thus, Phase II in mice typically contains several hybrid Phase I-II strokes, in contrast to rats, which move only a single forepaw [37]. Mice generally alternate between left and right paws in making Phase II strokes (though sometimes the same paw repeats a short series of Phase II strokes).

Phase III is highly visually distinctive, and consists of 1–5 large bilateral strokes with both paws. Both paws move very symmetrically almost as mirror images of the other, typically ascending together high up the side of the face, and passing forward synchronously over the ears. Phase III strokes are extremely stereotyped, usually all of the same height, and with both paws traveling back down to the nose between successive Phase III strokes [37]. The entire Phase III lasts 1–3 s.

Phase IV concludes the prototypical chain, and consists of a postural turn to the side and caudally, and lowering of the head to bring the tongue towards the flank or side of the body, followed immediately by a 2–5 s bout of body licking directed to the flank.

### Syntactic Initiation: rate of starting chains

**Figure 4:**
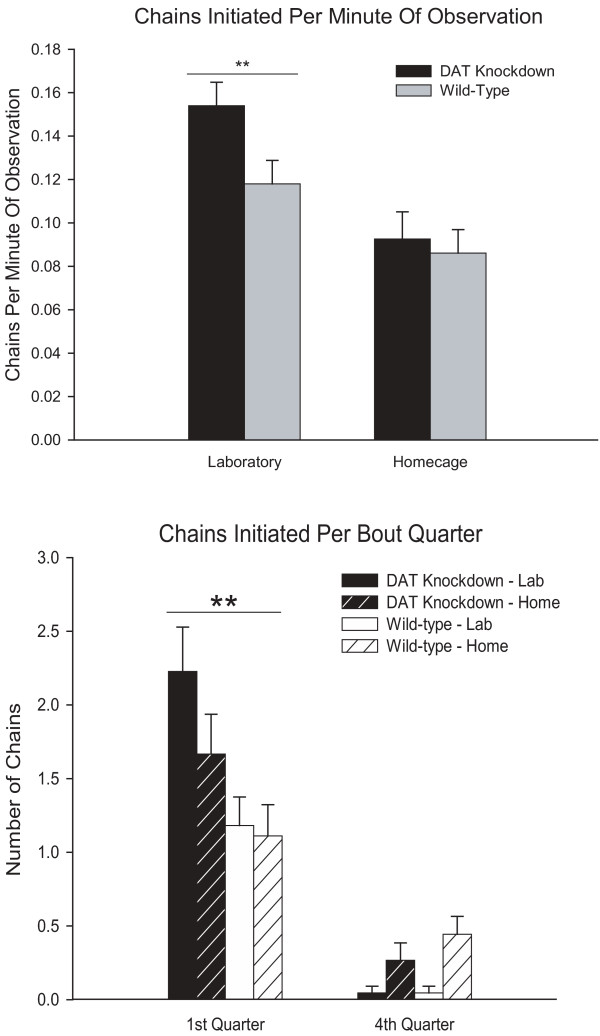
***Initiation of stereotyped syntactic chain pattern. ***Rates of initiation of syntactic chains are slightly higher for mutant mice, especially while grooming in the laboratory, measured cumulatively across the entire observation period (top). In more detail, initiation rates are broken down as occurring either early versus late in grooming bouts (bottom). All mice tend to start the stereotyped sequential pattern more often early in a grooming bout. Mutant mice are even more likely than wild-type mice to start the pattern in an early grooming bout, both in home and laboratory environments. ** p < 0.01.

In terms of the number of syntactic chains *started *during a grooming bout, *DAT-KD *mutant mice initiated marginally more syntactic chains overall than wild-type mice (F_(1,86) _= 3.592, p = 0.061; Figure 4). The difference in chain initiation was context dependent. All mice were twice as likely to initiate syntactic chains in the laboratory than at home (F(1, 82) = 85.73, p < 0.001), and mutant mice in particular initiated approximately 25% more chains than wild-type mice in the laboratory environment (F_(1,86) _= 17.315, p < 0.001; Figure 4), compared to only 5% more in the home environment. If the laboratory context was considered more stressful than the home cage environment, then stress dramatically promoted the tendency to begin a highly stereotyped sequence, especially for mutants.

The nature of the context-dependence of the difference was further clarified by a closer look at the time course of exactly when syntactic chains were begun by mice during a grooming bout. The overwhelming majority of syntactic chains tended to be initiated early in a grooming bout by all mice (Figure 4). Mutants initiated up to twice as many chains as wild-type mice per minute of grooming in the first quarter of a bout, whereas by the last quarter of their grooming bouts mutant and wildtype initiation rates no longer differed significantly (Figure 4). Mutants tended to begin more grooming bouts than wild-type mice especially in the laboratory (described below), which may have facilitated the mutants' greater tendency to initiate the syntactic chain pattern in the laboratory (Figure 4). In short, syntactic chains were initiated early in a grooming bout by all mice, but mutant mice were even more likely than wild-type mice to initiate chains during those early portions of a grooming bout, and the mutant advantage was greatest in the laboratory environment (which might be the most stressful environment).

### Syntactic completion

In mice and rats, once a syntactic chain pattern begins with Phase I, each remaining action can be predicted with roughly 80–90% accuracy. The entire syntactic chain occurs with a frequency over ten thousand times greater than could be expected by chance (based upon the relative probabilities of the component actions). However, several types or degrees of chain completion are possible.

### Types of syntactic chains

A prototypical or perfect syntactic chain requires Phases I, II, III and IV in order, with no deviations, additions or omissions. Perfect chains were occasionally performed by both mutant and wildtype mice. After performing Phase I, II and III strokes over the face, a mouse performs Phase IV by transitioning to body grooming. For this transition to body licking, the mouse must bend down and backward to bring its mouth toward a side flank, and then begin a vigorous bout of body licking that continues for 1–4 s.

In addition, several types of *imperfect syntactic chains *were observed in both mutant and wildtype mice. Imperfect sequences proceed from Phase I to IV with some minor deviation from the prototypical pattern along the way. In this study, we recognized three forms of imperfect completion. All involved a minor imperfection, which was either an insertion, reversal or replacement of a component action within the syntactic chain. Imperfect completion forms were: (i) *Reversal *of Phases II-III, where Phase II unilateral strokes were emitted after Phase III bilateral strokes (instead of before them), but the chain was otherwise syntactically correct; (ii) *Insertion *of an unexpected movement component in between phases, usually a quick paw lick or several paw licks inserted between Phases III and IV; (iii) *Skip or omission *of one phase en route to completion, where a chain lacked either any Phase II unilateral face stroke, or any Phase III face strokes (never both), but was otherwise syntactically correct (e.g. an observed order of I-III-IV). Finally, a fourth type of syntactic chain was observed that failed to be completed in the prototypical sense, but where the mice substituted paw licking in the terminal place of Phase IV (which might provisionally be regarded as an "attempt" to complete syntactically). We called this *Terminal substitution: *the final Phase IV component (body licking) was completely replaced with a different type of licking movement (paw licking), and the chain was otherwise syntactically correct (e.g. I-II-III-paw lick bout).

Terminal substitution never attains a prototypical Phase IV, and so is not really a form of syntactic completion by criteria used in earlier studies. However, the terminal substitution of paw licking might be viewed as an attempt to complete syntactically with a transition from paw strokes to licking, compared to other forms of incompletion such as either simply stopping or immediately launching into a sequentially flexible series of grooming strokes. Thus for the purpose of analysis, we examined the consequences of allowing terminal substitution to count provisionally as a form of "weak" completion.

At the completion of Phase IV (strong or weak), over 93% of syntactic chains led to continued grooming of body or face in sequentially flexible and much less predictable patterns compared to syntactic chains. After 7% of chains, the end of Phase IV terminated the entire grooming bout, and the mouse rested quietly afterwards or began to explore the chamber.

### Syntactic rigidity: strength of pattern completion

*DAT-KD *mutant mice not only started more syntactic chain patterns, they were also more likely than wild-type mice to complete the syntactic chain patterns they started – in both laboratory and home environments (Figures 2 &3). Sequential super-stereotypy (i.e. more predictable and stereotyped completion of entire sequence) of *DAT-KD *mutant mice was the most consistent and robust finding of our study (F_(1,78) _= 12.33, p < 0.001; Figure 2).

The higher syntactic rigidity of mutants was visible qualitatively and verified quantitatively (Figures 2 &3), and it interacted with the various types of syntactic completion described above (interaction between mutant/wildtype × perfect/imperfect types: F_(4,184) _= 5.96, p < 0.001). Hyper-dopaminergic mutant mice nearly always completed their syntactic chains with the strong form of Phase IV (body licking), whereas wild-type mice completed roughly half their chains with only the weaker form of Phase IV (paw licking). Mutant mice completed a higher percentage of insertion, reversal and omission types of syntactic chains than wild-type mice (F(4, 184) = 129.01, p < 0.001; each subtype; Figures 2 &3). These stronger or more rigid chains of mutant mice more closely corresponded to the prototypical 4-phase syntax pattern (including the prototypical terminal Phase IV component: body licking).

In other words, mutant mice were better than wild-type mice at resisting disruption of the pattern by minor flaws that occurred along the way, and mutants more often returned to the full-blown pattern after any distraction. For example, insertion chains included 1 or 2 extraneous movements, such as a nonsyntactic paw lick action inserted between Phases III and IV. After an insertion, mutants were nearly 50% more likely than wild-type mice to reach a strong form of Phase IV completion (mutant vs. wildtype, p < 0.01, Bonferroni). Similarly, a reversal error reversed the serial order of Phases II and III, or followed a Phase II stroke with a late Phase I ellipse stroke, and after a reversal mutant mice were nearly 50% more likely than wild-type mice to go on to complete a strong form of Phase IV. Finally, in an omission chain, a mouse would omit either Phase II or Phase III (never both), and after an omission mutants were again roughly 50% more likely than wild-type mice to successfully return to the full pattern and reach a strong form of terminal Phase IV completion (each p < 0.01, Bonferroni).

In contrast, wild-type mice had a greater proportion of terminal substitution chains that never achieved a full syntactic transition to body grooming. Wild-type mice instead substituted a weaker paw-lick form of Phase IV as terminal component. In terminal substitution, a mouse completely omitted the normal Phase IV shift to body licking, and instead simply continued to lick its paws, never changing posture or moving its head caudally out of the normal facial grooming position (the complete failure of transition to body licking after paw licking marked the difference between Insertion and Terminal Substitution chains). Wild-type mice had nearly twice the proportion of terminal substitutions as mutant mice (F(1,78)= 11.47, p < 0.001).

If terminal substitution is regarded as failure to complete the pattern, then wild-type mice simply failed to complete over half the syntactic chains they began. More leniently, wild-type mice could approach an 80% – 90% rate of syntactic completion – if we took the unprecedented step of allowing Phase IV terminal substitution to count as weak completion (Figure 2). Allowing this weaker criterion was the only way to consider wild-type mice able to achieve the 80%–90% syntactic completion level that mutant mice successfully achieved through the stronger prototypical form of Phase IV.

In summary, *DAT-KD *mutant mice had more rigid sequential patterns than wild-type controls in several ways. Mutant mice were more likely than wild-type mice to proceed syntactically through Phases I, II and/or III to reach the syntactic final Phase IV (body licking). Even after encountering minor imperfections along the way, mutants persevered in the sequential pattern. Wild-type mice introduced the same imperfections in their syntactic pattern, but did not return to the full pattern or complete Phase IV as strongly, ending their chains without ever reaching the full-blown transition to body grooming that normally terminates a syntactic chain pattern.

Finally, syntactic completion was highest in home environment grooming for all mice (even though more syntactic chains were begun in laboratory) (F(1,78)14.31, p < 0.001). This difference suggests that stress may promote the initiation of stereotyped sequences, but impede their lawful completion, and is consistent with reports that stress disrupts completion of syntactic chain sequences [62]. However, mutant mice were equally more likely than wild-type mice to complete strong patterns in both laboratory and home environments.

### Motor control for movement capacity

In order to reject motor confounds that might have provided an alternative explanation of some results, we assessed whether wild-type mice were simply less able to perform body licking movements than mutant mice. If wild-type mice had motor deficits that impaired their ability to perform body-lick posture/movements, then wild-types might have had weakened syntactic chains simply because of their motor incapacity to perform Phase IV movements, rather than because mutants had stronger sequencing tendencies. Therefore we analyzed whether wild-type mice spent a lower proportion of their total grooming behavior time making body licking movements compared to mutant mice. However, wild-type mice did not have significantly lower total cumulative duration scores for body licking overall than mutant mice (F(1,78) = 0.56, n.s.), indicating there was no motor impairment of Phase IV movements. That suggests the difference in tendency to complete syntactic chains represents a true difference in sequence rigidity or pattern strength, and not in simple motor capacity.

### Overall grooming behavior: amount, bout number, and bout duration

**Figure 5:**
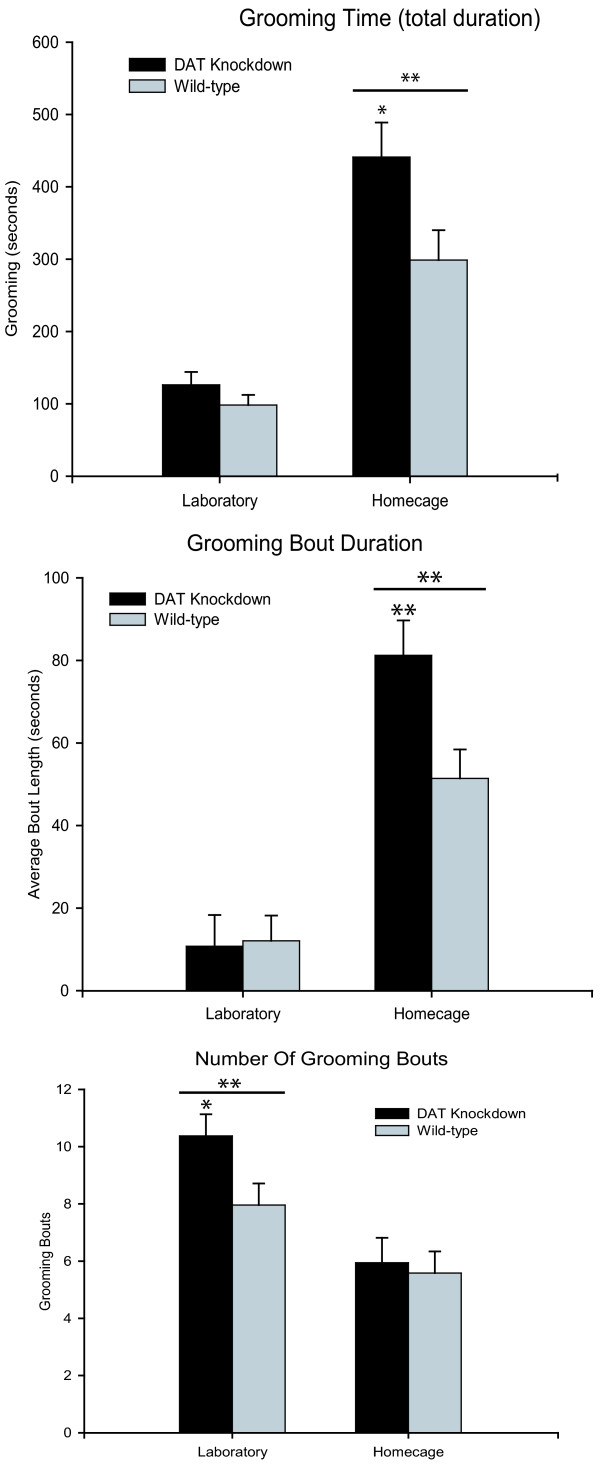
***General amount and bout features of grooming behavior. ***Cumulative time spent in grooming behavior during observation (total duration), Duration of individual bouts of grooming, and the Number of bouts of grooming emitted during observation session. Mutant mice tend to spend more time in grooming than wild-type mice, and to have longer grooming bouts, in the home environment. Mutant mice tend to emit a greater number of fragmented bouts when grooming in the laboratory environment. These general features of grooming enhancement in mutant mice are flexible and context-dependent, in contrast to the greater mutant rigidity of sequential pattern that is constant across both environments (shown in Figure 2). * p < 0.05; ** p < 0.01.

All mice groomed twice as much in their home cages than in the laboratory environment, suggesting that the relatively novel laboratory environment might have acted to suppress spontaneous grooming behavior (F_(1,82) _= 1.773, p < 0.001; Figure 5). Grooming behavior in the laboratory was less than half that of the home cage for both mutants and wildtypes (in terms of cumulative grooming duration per hour of observation). *DAT-KD *mutant mice spent 10%–50% more time than wild-type control mice in grooming behavior overall (F_(1,86) _= 3.949, p < 0.05), and the mutant propensity to groom more was most visible in the home environment (p < 0.1).

Closer analysis of grooming compared the relative contributions to increased grooming time of greater bout numbers versus longer bout durations (Figure 2). The increased time spent grooming by mutant mice in the home cage was due to longer grooming bouts (but not to a greater number of bouts) compared to wild-type mice. In their home cages, grooming bouts in mutant mice were 80% longer than in wild-type mice (F_(1,86) _= 4.083, p = 0.008), while bout numbers did not differ.

Although mutants emitted only marginally more body licking than wild-type mice in an analysis that combined data from both home and laboratory environments (F_(1,60) _= 3.403, p < 0.07), a separate analysis of grooming specifically in the home cage showed that mutants at home had longer cumulative durations of body licking (p < 0.05, Bonferroni), consistent with prolongation of the later components of cephalocaudal grooming bouts in that home environment [63]. However, as a percentage of total grooming, the proportion of mutant body licking to facial stroke components was not higher than for wild-type mice, either overall (F(1,60) = 0.58, n.s.), or even in the home cage (p = 0.32), which suggests that the mutants' longer grooming bouts in the home cage may also have included more facial strokes than wild-type mice. Thus, longer mutant grooming bouts in the home cage likely involved expansion of several components of grooming, including longer body licking bouts and facial strokes. These perseverative features of *DAT-KD *mutant grooming in the home cage therefore may overlap with perseverative body grooming tendencies reported for other genetic animal models of compulsive behavior, such as *Hoxb8*^*lox *^and *D1CT *mutant mice [13,51-53,55,57].

Conversely, in the laboratory environment, *DAT-KD *mutants' higher grooming was chiefly due to a greater number of grooming bouts (but not longer bouts). In the laboratory environment, mutants began more grooming bouts than wild-type mice (F(1,86) = 3.478, p = 0.026), but their duration of bouts did not differ. Thus, different features of grooming bouts (length versus number) were enhanced in mutant mice depending on their environmental context of the moment. However, as described above, in both home and laboratory the hyper-dopaminergic mutants were always more likely than wild-type mice to perform more rigid and strongly stereotyped syntactic chain sequences.

## Discussion

### Sequential super-stereotypy: pattern completion

Our results reveal that hyper-dopaminergic mutant mice show excessively strong and rigid manifestations of a complex fixed action pattern compared to wild-type mice. Their sequential super-stereotypy was produced by DAT knockdown mutation, which reduces DAT to 10% of wild-type levels and causes extracellular dopamine elevation to 170% in neostriatum [58]. Mutant mice showed more stereotyped and predictable syntactic grooming chains, the instinctive fixed action pattern that serially links up to 25 movements into 4 predictable phases that follow 1 syntactic rule. That entire pattern became even more stereotyped and resistant to disruption in hyper-dopaminergic mutant mice.

The stronger pattern was evident in several ways. First, *DAT-KD *mutant mice were more likely to begin a syntactic chain pattern than wild-type mice, especially during the early minutes of a grooming bout (when the highly stereotyped serial pattern is most likely to be produced), and especially in the novel laboratory environment (a potential stressor). Further, once the complex sequence began, *DAT-KD *mutant mice went on to execute chains that were more stereotyped and rigid, both qualitatively and quantitatively. Qualitatively, mutant mice almost always achieved the strongest form of the terminal phase (Phase IV), successfully making a transition from head grooming to body grooming. By comparison, wild-type mice ended far more of their chains with a weaker terminal substitution for Phase IV, which left them stuck in head grooming without ever making a transition to body grooming. Quantitatively, *DAT-KD *mutant mice returned more often to the prototypical pattern after minor mistakes, whereas wild-type mice failed to reach full Phase IV after such mistakes. Mutant mice returned more often to the full pattern after extraneous component insertion, phase omission, or serial reversal of phases. The mutants' elevated pattern strength for this complex sequence was evident in both home and laboratory environmental contexts.

If the less-stereotyped sequential patterns of wild-type mice are viewed as the norm (and not as a sequential deficit), then the mutant tendency to complete stronger syntactic patterns must be viewed as *sequential super-stereotypy*, representing the exaggerated serial rigidity feature of compulsive behavior. Here sequential super-stereotypy is manifest in a complex behavioral sequence that is instinctive and naturally stereotyped to begin with, but becomes even more stereotyped or excessively rigid as a consequence of the DAT mutation.

It may be important that the mutant pattern strength is revealed not as an elimination of errors, but rather primarily as a resistance to disruption by errors. In other words, mutants did not have more frequent perfect chains than wild-types: both generated similar moderate rates of minor errors (e.g., inserting extra actions, omitting one syntactic phase from where it ought to be, or reversing the order of 2 phases in the 4-phase pattern). Instead, the mutants' stronger syntactic pattern was like a tightened elastic band, pulling them back after such errors to finish the prototypical pattern. Stronger return to the pattern could only be possible if DAT knockdown strengthened the entire pattern as a global whole, facilitating the mutants' ability to maintain a neural representation of the pattern during an error and to resume the remaining pattern after the error. That suggests that neural mechanisms of pattern coordination were better able to persist in rule maintenance in the face of disruption, and to successfully compete to re-establish control of the behavioral stream after the disruption.

Thus, stronger patterns were not simply the results of strengthened Markov sequential transitions among individual pairs of actions, producing a stimulus-response (S-R) reflex chain. If sequence composition was simply a probabilistic construction based only on the frequency of transitions between individual pairs of actions, then stronger perfect completion might have been expected in mutants, but not stronger return after an error. Errors would still terminate or weaken the pattern. Instead, we observed the opposite result: mutants kept errors but recovered better after them, and took the full pattern up again where it had left off.

### Relation to other nigrostriatal manipulations and behaviors

These results are the first demonstration to our knowledge of sequential super-stereotypy of a complex behavioral pattern, occurring spontaneously without drugs. In previous studies, dopamine D1 agonists were needed to cause sequential super-stereotypy of syntactic grooming chains, whereas D2 agonists in contrast reduced initiation and completion of syntactic grooming chains (even though D2 agonists can cause simple repetitive movement stereotypies) [43-45,64]. Future studies will be needed to confirm whether the sequential super-stereotypy of DAT knockdown mutant mice depends specifically on increased D1 receptor activation. However, it is notable that there is a consistent trend of D1 circuit-activation inducing OCD-like behavioral persistence in D1 agonist-treated rodents, D1-circuit potentiated *D1CT *mice, and hyperdopaminergic *DAT-KD *mice [43-45,53,55,57,64]. This suggests that the D1 circuit may play an important role in features of compulsive behavior related to perseveration and sequential rigidity. It also would be of interest for future studies to examine if other animal models of perseverant grooming behavior, such as *Hoxb8*^*lox *^and *D1CT *mutant mice, also show any exaggerated serial rigidity features in their fixed action patterns similar to those found here [13,51,53-55,57,65]. Finally, it would clearly be of interest to examine whether any other instinctive fixed action patterns belonging to those of DAT-KD mutant mice show sequential super-stereotypy similar to syntactic grooming chains.

We should note that although our study is the first to produce spontaneous sequential super-stereotypy, several previous studies reported *weakening *of the syntactic chain pattern by other genetic manipulations. For example, the ability to complete syntactic grooming chains is impaired in several types of mutant mouse, caused by either a knockout of D1 dopamine receptors [41], or by a Weaver gene mutation that alters the nigrostriatal dopamine system [65,66]. In the D1 knockout study, mutant D1 mice were less able than wild-type mice to complete the full grooming pattern of syntactic chains they started [41]. Our *DAT-KD *findings provide an opposite demonstration to complement that D1 knockout study: DAT knockdown *strengthens *the same pattern presumably by elevating extra-cellular dopamine. Both results might therefore reflect essentially linear effects on the sequential stereotypy of this complex behavior pattern, mirroring up or down changes in basal ganglia dopamine neurotransmission.

### Evolution co-opts sequential super-stereotypy

We acknowledge that there is one other known form of genetically-related sequential super-stereotypy for syntactic grooming chains. However, that sequential super-stereotypy is not caused by a single targeted gene mutation but rather is a naturally evolved adaptation of the fixed action pattern in a species of ground squirrel, *Spermophilus beecheyi *[38], which is probably polygenic in origin. California ground squirrels defend their individual mating territories in the Sierra mountains against other same-sex ground squirrels (especially males against other males). One of their behavioral territory displays is a specialized exapted form of the syntactic grooming chain [38].

Display forms of *Spermophilus beecheyi *syntactic chains are ritualized, more sequentially rigid and predictable than normal self-grooming chains, and occur as a single grooming chain with no other grooming before or after [38]. Phase III elements are amplified and made more visually distinctive, and an extra Phase V component is appended to the end of the pattern (the squirrel seizes and licks its tail, which is also visually distinctive). Syntactic grooming chains are usually performed at the boundary where two adjacent territories meet. Syntactic grooming chain displays appear to be communicative, in that they are emitted in conjunction with other territorial displays, such as scent-marking of objects, and have the social consequence of subsequently reducing the likelihood of a physical fight between the two adversaries [38].

Thus the evolution of *Spermophilus beecheyi *ground squirrels appears to have exapted the pre-existing pattern of a syntactic grooming chain, which likely evolved in ancestral rodents over 60 million years ago, and co-opted it into a sequentially super-stereotyped form for specific communicative use [38]. It may have been selected because of the same feature that led us to study syntactic chains, namely its recognizable sequential stereotypy. Also, the sequential pattern appears highly sensitive to the underlying genotype; for example, the detailed 'signature patterns' of the syntactic grooming chains that distinguish mice from squirrels, rats, guinea pigs and other rodents can be used to construct taxonomic trees of relatedness for them (similar to taxonomies based on differences in skull structure or in DNA sequences) [37]. The genetic sensitivity of the pattern may explain why evolutionary selection exploited it for use by California ground squirrels, and also explain why knockdown of a single gene can change the strength of the entire complex sequential pattern in studies such as ours [41,65,66].

### Neural systems and clinical implications of sequential super-stereotypy

Altered neurochemical signaling within basal ganglia neural circuits may be the mechanism by which DAT knockdown produces sequential super-stereotypy of grooming syntax. Electrophysiological studies have shown that neurons in neostriatum and in substantia nigra pars reticulata code the sequential pattern of syntactic grooming chains and other natural sequences of behavior [24,27,28]. For example, 40% of neostriatal neurons in rats code sequential aspects of the syntactic chain pattern, especially in anterior dorsolateral neostriatum [24,27]. Neurochemical boosting of dopamine signalling from substantia nigra pars compacta on to neostriatal neurons might be one candidate mechanism to modulate sequential super-stereotypy of the pattern in *DAT-KD *mutants. Similarly, neurons in the substantia nigra pars reticulata appear especially to code initiations of the complex behavioral sequence, and so modulated input to them might be more relevant to the elevated mutant tendency to begin the syntactic pattern [24,28].

Nigrostriatal mechanisms for sequencing instinctive action may have been co-opted in subsequent mammalian and human evolution into use in sequencing learned and cognitive psychological elements [67-69]. In that way, the same basal ganglia mechanisms used for movement syntax may participate in sequential habits that result from learning [20,29,70-72]. A view of basal ganglia as a general purpose sequencing mechanism is compatible also with computational sequencing models of basal ganglia [16-19]. Beyond the basal ganglia, *DAT-KD *mutant mice might also have elevated extra-cellular dopamine concentrations in other target structures, including prefrontal cortex and amygdala. Such systems might also contribute to OCD and Tourette's syndromes in humans and to some aspects of compulsive-like behavior in mutant mice. Elaborated applications of dopamine-related circuits for sequencing may thus extend from instinctive animal behavior to abstract human cognition and behavior, including syntactic sequencing of action plans, linguistic syntax, and the serial order of streams of thought [14,73].

A clinical implication of the embeddedness of basal ganglia in sequencing function may be a vulnerability to sequential dysfunction in some human disorders involving nigrostriatal systems [74,75]. Both Tourette's syndrome and obsessive-compulsive disorder show symptoms of sequential super-stereotypy, in the form of overly rigid patterns of action, language or thought [76,77]. Basal ganglia are believed to be involved in generating such pathologically-strong and complex sequential stereotypies [1,2,8,9,74,78-85]. Hyper-dopaminergic function in nigrostriatal and related neural systems might thus play a role in causing the excessive rigidity of behavioral tics, repetitive language utterances, and obsessive chains of thought [2,74,79,81,86,87].

Finally, while highly speculative, it is at least conceivable that an evolutionary specialization of dopamine-related neural mechanisms for self-grooming sequences, suggested by our current results, might also influence the theme or content, as well as the syntactic stereotypy, of some human super-stereotypies involving washing or purifying compulsions [74].

Pathologically-intense rituals of cleanliness, security behavior, or concerns with contamination, all share a focus that might relate to grooming of oneself [74]. Conceivably, excessive activation in brain circuits linked by evolution to self-grooming behavior might tip the thematic focus of some human stereotyped sequences toward rituals of cleanliness or reaction to perceived contamination, in addition to strengthening their syntactic rigidity. Whether or not such a direct overlap exists between human pathology and animal instinctive behavior, our results indicate that *DAT-KD *mutant mice show sequential super-stereotypy in a complex instinctive fixed action pattern.

## Methods

### Subjects

*DAT-KD *mutant mice (n = 12 male) and wild-type control mice (n = 12 male) were generated at the University of Chicago by breeding heterozygous mutants on a 129 Sv/J genetic background as described earlier [58]. Such a design minimizes any contribution to behavioral phenotype by genetic background difference or by differences in genetic modifiers that are linked to the *Slc6a3 *locus. DAT knockdown was achieved by insertion of the tetracycline regulatable system into the 5' untranslated region in the second exon of the DAT gene (Slc6a3). Such an insertion reduced the DAT promoter strength without affecting its expression pattern. It also allows regulation of DAT expression by dietary tetracycline, although that feature was not used in this study. DAT knockdown reduces adult DAT expression to 10% of wild-type levels and raises extracellular dopamine levels in neostriatum to 170% (wild-type control = 100%) [58]. Once housed at the University of Michigan, mutant and wild-type mice (age 2–4 months) were allowed to habituate to their new surroundings for two weeks before any behavioral testing. Mice were housed at ~21°C on a 12 h light/dark cycle with lights on at 7 a.m., in groups of two to three same-type individuals during the laboratory environment testing phase. During the home cage testing phase of the experiments, mice were housed individually to facilitate videotaping. Food (Purina Rat Chow; St. Louis, MO) and water (tap water) were always available.

### Behavioral testing

It was important to determine whether any sequential stereotypy difference between mutant and wild-type mice in grooming behavior was a stable difference in action syntax strength, and not merely an artifact of testing conditions. Grooming behavior of rodents is sensitive to environmental contexts, both in quantity and in fine structure, and stressors in particular can either suppress or increase grooming behavior depending on type [88]. All mice were therefore tested for grooming behavior in 2 environmental contexts: 1) a standard behavioral neuroscience laboratory chamber, and 2) their own home cages (a relatively stress-free environment).

### Laboratory environment

Immediately prior to testing, mice were transported in their home cage on a cart down a 30 m hallway to a laboratory testing room with standard white fluorescent lighting, and placed individually in a test chamber (light intensity 550–650 lux; sound intensity 65–70 decibels measured within chamber). The laboratory test chamber consisted of a transparent cylinder (19 cm high, 12.5 cm diameter) suspended over a tilted mirror. A camera lens focused on this mirror gave a close-up view of the mouse's face, forepaws, and upper body. For behavioral testing, each mouse was placed individually in a test chamber and videotaped for 30 minutes. Each mouse received 3 habituation days in the laboratory test procedure before grooming behavior data were collected over the next 2 consecutive days in 30 min sessions.

### Home environment

Testing in the home environment took place during the dark phase under dim red light conditions. Mice were housed singly in transparent rectangular cages (12 cm high × 19 cm long × 10 cm wide). Videotaping of grooming sequences took place from the side of the transparent home cage, for 30 min each day on 2 consecutive days, with the camera focused closely on the mouse.

### Behavioral video analysis

Videotaped grooming behavior was scored in slow motion (frame-by-frame to 1/10^th ^actual speed; scorer blind to genotype) for grooming amount (cumulative durations), grooming bout number and bout length, and occurrence of syntactic chains. Syntactic grooming chains were identified and classified in frame-by-frame analysis as either Perfect, Imperfect but completed by full Phase IV (omission, insertion, or reversal types), Terminal substitution of paw lick for Phase IV body licking, or Incomplete (grooming stops before Phase IV, or reverts to sequentially flexible facial grooming and paw strokes) [37,41], [43,44]. We also made choreograph diagrams of syntactic chains from each mouse to compare details of their form and sequential pattern [61]. Behavioral data were statistically analyzed by 3-factor, 2-factor, or 1-factor ANOVA as indicated above. When significant results were obtained, post hoc paired comparisons were subsequently performed using Bonferroni or Tukey tests (alpha set equal to original 0.05 level).

## Authors' contributions

KCB conceived and supervised the study and drafted the manuscript; JWA co-conceived the study and participated in interpretation and writing; KRH carried out behavioral testing, videoanalysis, and statistics; XZ developed and generated the mutant mice, and participated in writing the manuscript.

## Supplementary Material

Additional File 1 ***Movie: Sequential super-stereotypy of an instinctive fixed action pattern in hyper-dopaminergic mutant mice. ***Windows Media Player movie file (.avi): *DAT Knockdown grooming fixed action pattern.avi*Examples of syntactic grooming chains performed by three hyperdopaminergic mutant mice are shown in the accompanying movie file. Choreograph diagrams of component movements' form and sequence are displayed for each syntactic chain, and strokes are illuminated sequentially in synchrony with their corresponding movements. Note that the first two syntactic chains contain insertion or reversal errors (Mutant mouse 1: paw lick insertions in Phase II, between Phases II and III, and between Phases III and IV; also reversal insertion of a Phase I ellipse stroke within Phase II. Mutant mouse 2: paw lick insertions within Phase I, within Phase III, and between Phases III and IV). However, the syntactic chains are not disrupted by these errors, and the mutants continue on with the sequential pattern to successfully complete Phase IV (body licking). Mutant mouse 3 also shows the ventral view that permits the viewer to see both forepaws simultaneously, which was used to score all syntactic chains in the laboratory. Click here for file
